# A Comparative Transcriptomic Analysis of Human Placental Trophoblasts in Response to Pathogenic and Probiotic *Enterococcus faecalis* Interaction

**DOI:** 10.1155/2021/6655414

**Published:** 2021-01-28

**Authors:** Qianglai Tan, Zhen Zeng, Feng Xu, Hua Wei

**Affiliations:** ^1^Xiamen Medical College, Xiamen 361023, Fujian, China; ^2^State Key Laboratory of Food Science and Technology, Nanchang University, Nanchang 330047, Jiangxi, China

## Abstract

With the ability to cross placental barriers in their hosts, strains of Gram-positive *Enterococcus faecalis* can exhibit either beneficial or harmful properties. However, the mechanisms underlying these effects have yet to be determined. A comparative transcriptomic analysis of human placental trophoblasts in response to pathogenic or probiotic *E. faecalis* was performed in order to investigate the molecular basis of different traits. Results indicated that both *E. faecalis* Symbioflor 1 and V583 could pass through the placental barrier *in vitro* with similar levels of invasion ability. In total, 2353 (1369 upregulated and 984 downregulated) and 2351 (1233 upregulated and 1118 downregulated) DEGs were identified in Symbioflor 1 and V583, respectively. Furthermore, 1074 (671 upregulated and 403 downregulated) and 1072 (535 upregulated and 537 downregulated) DEGs were only identified in Symbioflor 1 and V583 treatment groups, respectively. KEGG analysis showed that 6 and 9 signaling pathways were associated with interactions between Symbioflor 1 and V583. GO analysis revealed that these DEGs were mainly related to cellular and metabolic processes and biological regulation. However, 28 and 44 DEGs were classified into terms associated with placental and embryonic development in Symbioflor 1 and V583 treatment groups, respectively. Notably, 9 and 25 unique DEGs were identified only in Symbioflor 1 and V583 treatment groups, respectively. A large proportion of transcriptional responses differed when compared between pathogenic and probiotic *E. faecalis* interaction, and several unique DEGs and signal pathways were identified in the two different groups. These data enhance our understanding of how different traits can be affected by pathogenic and probiotic *E. faecalis* and the mechanisms underlying these effects.

## 1. Introduction

*Enterococcus faecalis* is a Gram-positive bacterium that is commonly found in a variety of different matrices including the alimentary tract and foods. This bacterium has received substantial attention due to the fact that it can exert both beneficial and pathogenic effects [1]. Certain *E. faecalis* strains are regarded as commensal bacteria or even probiotics for creating a healthy gut environment; however, other strains are considered to be dangerous as they can lead to a variety of nosocomial infections and diseases [2, 3]. Previous researches have shown that *E. faecalis* is able to pass through biological barriers and can subsequently mediate a variety of effects in the host [4, 5]. A review by Goldenberg et al. confirmed that various enterococci could transmit to fetus and cause stillbirth [6]. We have previously demonstrated that *E. faecalis* OG1RF can pass through the placental barrier of pregnant mice, translocate into the fetus, and then affect both fetal and placental growth and development [7]. We demonstrated that *E. faecalis* OG1RF induces placental and embryonic development retardation, stress and stimulus response activation, apoptosis, immune response disorder, and cell adhesion disintegration of placental trophoblasts through various signaling pathways using real-time PCR and DNA microarray [8]. *E. faecalis* has also been identified in meconium obtained from healthy neonates. The presence of this type of bacteria in meconium could initiate gut colonization as an adaptation to the fetal gut to prepare the fetus for life outside the mother [9]. Albesharat et al. found that *E. faecalis* was present in the feces of mothers and their babies and hypothesized that the initial bacterial colonization of the infant occurs via vertical transmission from mothers to infants [10]. The close relationship between two typical *E. faecalis* strains (Symbioflor 1 [11] and V583 [12]) has been studied in significant detail with regards to human pregnancy. *E. faecalis* Symbioflor 1 (SymbioPharm, Herborn, Germany) is recognized as a probiotic strain that can alleviate the symptoms of irritable bowel syndrome by improving the microbial balance in the intestine. *E. faecalis* V583 is a vancomycin-resistant prototype clinical isolate that causes opportunistic nosocomial infection worldwide. Meanwhile, a human placental choriocarcinoma BeWo cell lines, a widely recognized *in vitro* placental model [13], was adopted. The BeWo cell line is regularly used to study a range of placental functions, including transplacental transport and infection caused by viruses and bacteria. DEGs of BeWo cells associated with *E. faecalis* invasion were identified by using DNA microarray, and transcriptomic profiling was compared by using Gene Ontology (GO) and Kyoto Encyclopedia of Genes and Genomes (KEGG) pathway database [14].

In this study, we first constructed an interaction model between two different *E. faecalis* strains and BeWo cells. Then, we compared the invasion abilities (adhesion ability and internalization ability) between the two *E. faecalis* strains. Subsequently, we performed comparative transcriptomic profiling by using a DNA microarray (Illumina Human HT-12 v4 Expression Bead Chip), GO analysis, and KEGG pathway analysis. The main objective of this study was to gain insights into the differences in transcriptional regulation between strains of *E. faecalis* that can exhibit dualistic behavior towards host health. The study was designed to identify the different traits that might be affected by putative pathogenic or probiotic *E. faecalis*. The identification of such traits is crucial as this information may ultimately contribute to the future development of strategies for the prevention and treatment of invasion and infection caused by different *E. faecalis* strains.

## 2. Materials and Methods

### 2.1. Bacterial Strains and Cell Lines

The bacterial strains used in this study are listed in Table 1. In brief, strains of *E. faecalis* strains, clinical pathogen V583 (ATCC 700802), and probiotic Symbioflor 1 (DSM 16431) were cultured in Trypticase soy broth (TSB) at 37°C for 24 h with shaking at 180 rpm. The bacteria were then harvested by centrifugation at 6000 rpm for 10 min, washed twice with sterile PBS (0.01M, pH 7.4), and reconstituted in cell culture medium DMEM/F-12 (Solarbio, Beijing, China) to yield a concentration of 10^9^ CFU/mL before use.

The human choriocarcinoma cell line BeWo was purchased from Action-award Biotech Co., Ltd. (Guangzhou, China) and cultured as described previously [8]. In brief, BeWo cells were cultured in DMEM/F-12 medium supplemented with 10% (v/v) FBS (Gibco, Grand Island, NY) at 37°C under a 5% CO_2_ atmosphere, until approximately 80%–90% cell confluence.

### 2.2. Invasion Assays

Invasion assays were performed in accordance with our previous study [8]. BeWo cells were transferred into 24-well plates (Corning, NY) and cultured for 24–48 h until a confluent monolayer was obtained. A 5 *μ*L aliquot of resuspended bacteria was then added to each well containing 495 *μ*L of cell culture medium and incubated for 60 minutes.

The total number of invading bacteria was determined by twice dip-washing with sterile PBS to remove free bacteria and lysing the BeWo cells with 500 *μ*L of 0.5% Triton X-100/PBS to release the internalized bacteria. Serial dilutions were spread onto Trypticase soy agar (TSA) plates and incubated at 37°C overnight. The numbers of internalized bacteria were then determined by adding 100 *μ*g/mL of gentamicin and 50 *μ*g/mL of penicillin and incubating for 60 min to kill any viable extracellular bacteria that were still present. The cells were then washed twice, lysed, and the bacterial count determined as described above. *P* values were calculated using Studentʼs *t*-test.

### 2.3. DNA Microarray and Data Analysis

Incubation experiments were performed as described in our previous study [8]. In brief, BeWo cells were grown in 24-well plates and cultured for 24–48 h until 80%–90% confluency. *E. faecalis* V583 and Symbioflor 1 were then harvested and resuspended in DMEM/F-12 cell culture medium to 10^9^ CFU/mL. A 5 *μ*L aliquot of resuspended *E. faecalis* V583 and Symbioflor 1 was then added to each well containing 495 *μ*L of cell culture medium and incubated for 4 h. The same amount of cell culture medium, but without any *E. faecalis* strains, was used as a negative control. Total RNA was extracted using TRNzol Total RNA Reagent (TIANGEN, Beijing, China). DNA microarrays were performed by Beijing EMTD Technology Development Co., Ltd. using an Illumina Human HT-12 v4 Expression BeadChip system (Illumina, Inc., San Diego, CA). In brief, RNA was adjusted to a concentration of 200 ng/*μ*L, followed by first and then second strand cDNA synthesis. Double-stranded cDNA was then purified with a filter cartridge, and cRNA was synthesized by T7 RNA polymerase transcription *in vitro*. Following purification and quantification, the cRNA was hybridized with the BeadChip, washed, scanned, and analyzed. Illumina expression data were deposited in the NCBI Gene Expression Omnibus (GEO) database under the accession number GSE75626. DEGs were analyzed using GO and KEGG pathway databases.

### 2.4. Statistical Analysis

Unless specified, all experiments were performed in triplicate. All data were analyzed using statistics programs contained in SigmaPlot 11.0 (Systat Software, San Jose, CA).

## 3. Results

### 3.1. Invasion Ability of *E. faecalis*

**Figure 1:**
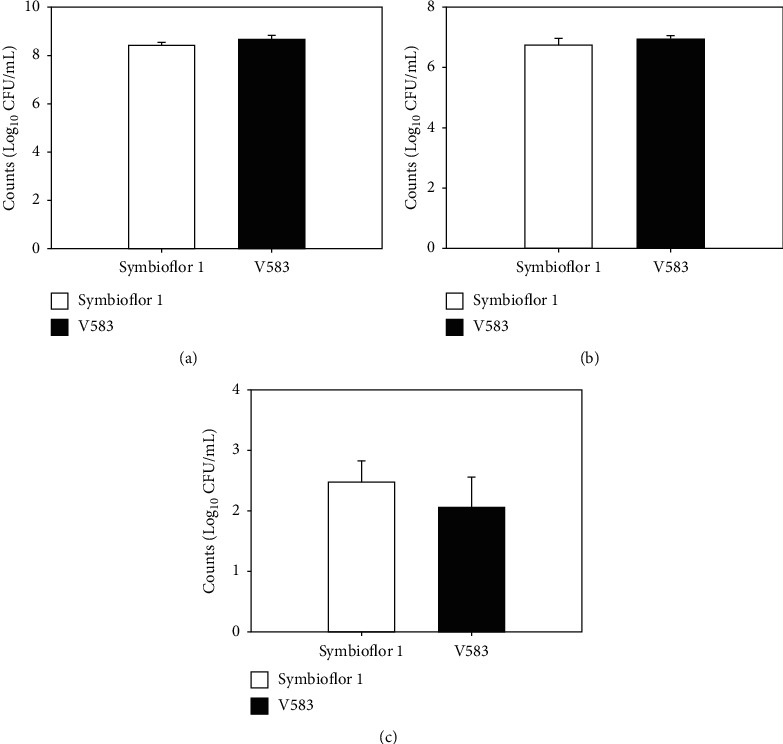
Invasion ability of *E. faecalis* strains associated with BeWo cells. (a) Colony forming units (CFUs) of *E. faecalis* strains after incubation; (b) CFUs of invaded *E. faecalis*; (c) CFUs of internalized *E. faecalis*. Values represent mean ± SD. *P* values were calculated using Student's *t*-test.

The number of *E. faecalis* strains present in BeWo cells was determined using invasion assays. As shown in Figure 1(a), 8.42 ± 0.13 Log_10_ CFU/mL of Symbioflor 1 and 8.66 ± 0.18 Log_10_ CFU/mL of V583 were identified in BeWo cells after incubation under the same conditions. These results showed that incubation conditions did not cause any deviations in the detection of invasion ability.

As demonstrated in Figure 1(b), the invaded cell counts of Symbioflor 1 and V583 strains were 6.76 ± 0.20 and 6.86 ± 0.20 Log_10_ CFU/mL, respectively, while those of internalized Symbioflor 1 and V583 were 2.48 ± 0.35 and 2.06 ± 0.50 Log_10_ CFU/mL, respectively (Figure 1(c)). These results showed that there was no significant difference in the *in vitro* invasion ability when compared between the two strains (*P* > 0.05). These data indicated that both the pathogenic and probiotic *E. faecalis* strains could pass through the placental barrier. Similar observations were noted with regards to the *in vitro* invasion ability when compared between the two strains.

### 3.2. Differential Gene Expression Profile Analysis

**Figure 2:**
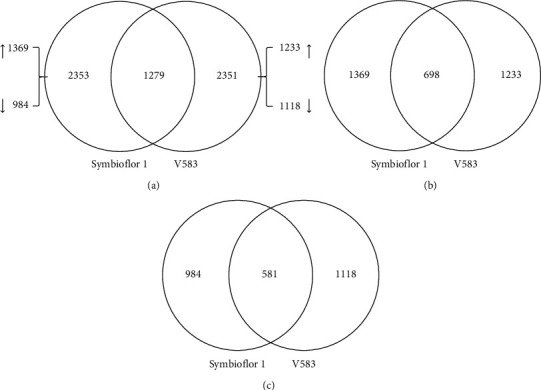
Distribution of DEGs of BeWo cells in response to different *E. faecalis* strains. Venn diagram showing the number of unique and common DEGs in BeWo cells in response to Symbioflor 1 and V583 infection. (a) Total DEGs; (b) upregulated genes; (c) downregulated genes.

DNA microarray techniques were used to compare the gene expression patterns of untreated BeWo cells with those infected by *E. faecalis*. After applying cutoffs for induction (ratio > 2.0-fold) and suppression (ratio < 0.5-fold), out of a total of 47,231 genes on the BeadChip, a total of 2353 DEGs, including 1369 upregulated genes and 984 downregulated genes were identified in the Symbioflor 1 treatment group. In the V583 treatment group, a total of 2351 DEGs, including 1233 upregulated genes and 1118 downregulated genes, were identified. Furthermore, 1279 of the total number of DEGs were found to be common to both treatment groups, thus accounting for 54.36% and 54.40% of the DEGs in Symbioflor 1 and V583 treatment groups, respectively (Figure 2(a), Tables S1 and S2).

In addition, 698 of the total number of upregulated DEGs were found to be common to both strains, thus accounting for 50.99% and 56.61% of the DEGs in the Symbioflor 1 and V583 treatment groups, respectively (Figure 2(b)). Furthermore, 581 of the downregulated DEGs were found to be common to both strains, thus accounting for 59.04% and 51.97% of the DEGs in the Symbioflor 1 and V583 treatment groups, respectively (Figure 2(c)).

In general, both pathogenic and probiotic *E. faecalis* treatment groups showed a similar number of total DEGs. However, the number of upregulated and downregulated genes varied significantly in both groups. Hence, DEGs were then mapped using the KEGG database for signal pathway analysis to gain further understanding of their biological function.

### 3.3. Signal Pathway Analysis of DEGs

According to signal pathway analysis of DEGs, six main terms associated with BeWo cells in response to invasion by Symbioflor 1, including the MAPK signaling pathway, Jak-STAT signaling pathway, adherens junction, T cell receptor signaling pathway, p53 signaling pathway, and pathogenic *Escherichia coli* infection. For the V583 treatment group, nine main terms were associated with the response of BeWo cells to invasion, including the MAPK signaling pathway, leukocyte transendothelial migration, p53 signaling pathway, T cell receptor signaling pathway, apoptosis, ErbB signaling pathway, adherens junction, B cell receptor signaling pathway, and pathogenic *Escherichia coli* infection. According to the results obtained as shown in Table 2, five common terms were identified in both treatment groups. However, the Jak-STAT signaling pathway in particular was only observed in the Symbioflor 1 treatment group, whereas the ErbB signaling pathway, apoptosis, B cell receptor signaling pathway, and leukocyte transendothelial migration were all identified in the V583 treatment group.

### 3.4. Bioinformatic Analysis of DEGs

DEGs were characterized functionally by comparison against GO database and classified into three different categories, namely, the biological process, cellular component, and molecular function. For the Symbioflor 1 treatment group, DEGs were categorized into 293 terms in the biological process, 58 terms in cellular component, and 70 terms in molecular function (Table S3). For the V583 treatment group, the DEGs were categorized into 267 terms in the biological process, 59 terms in cellular component, and 62 terms in molecular function (Table S4). According to the comparative analysis of relevant data from both treatment groups, the top three genes were classified under the GO biological process categories related to the same terms used for the cellular process, metabolic process, and biological regulation (Tables S3 and S4).

In particular, for the Symbioflor 1 treatment group, a total of 28 genes were classified into terms associated with placental and embryonic development: placental development, embryonic placental development, in utero embryonic development, and the embryonic process involved in female pregnancy (Table 3). However, for the V583 treatment group, a total of 44 genes were classified into terms associated with placental and embryonic development, namely, placental development, chordate embryonic development, in utero embryonic development, embryonic development ending in birth or egg hatching, embryonic cranial skeleton morphogenesis, and the embryonic process involved in female pregnancy. According to the results obtained (Table 3), 19 DEGs were found to be common to both treatment groups.

**Figure 3:**
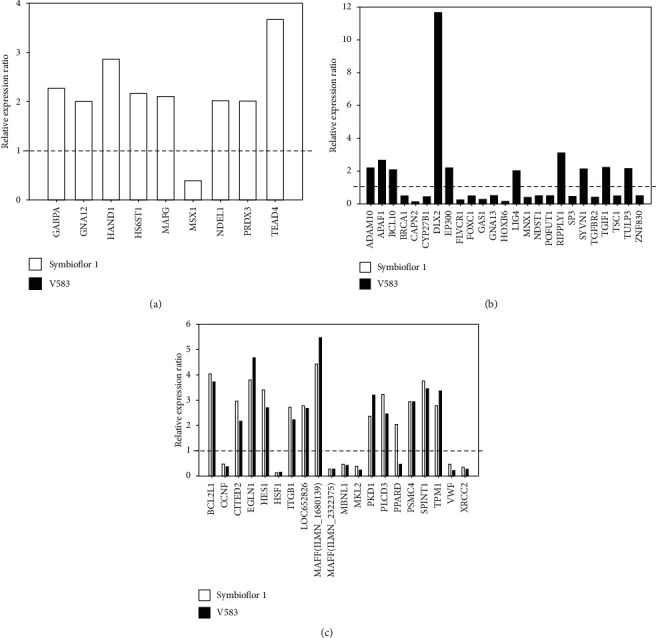
Fold change of DEGs classified into placenta and embryonic development. (a) Unique DEGs in the Symbioflor 1 treatment group; (b) unique DEGs in the V583 treatment group; (c) common DEGs in the Symbioflor 1 and V583 treatment groups. The relative expression ratio for each gene is presented in the histogram. A ratio greater than zero (>1) indicates upregulation of gene expression, and a ratio below zero (<1) indicates downregulation.

In addition, the fold-changes for these DEGs are shown in Figure 3. For the Symbioflor 1 treatment group, eight out of nine unique DEGs were upregulated, while one gene (*MSX1*) was downregulated. For the V583 treatment group, 10 out of 25 unique DEGs were upregulated, while 15 genes were downregulated. Furthermore, the most common DEGs showed a similar tendency to vary in each of the treatment groups. However, the *PPARD* gene in particular differed from all other genes as it was upregulated in Symbioflor 1 and downregulated in V583.

## 4. Discussion

In our previous studies, we demonstrated the ability of *E. faecalis* OG1RF to translocate both intestinal and placental barriers and demonstrated the molecular mechanisms responsible for these actions by DNA microarray analysis [7, 8]. However, the influence of *E. faecalis* in human pregnancy has not been fully elucidated, as different *E. faecalis* strains are expected to exhibit multiple roles. The objectives of the present study were to investigate and compare the different influences of putative pathogenic or probiotic *E. faecalis* on human placental trophoblast cells. Hence, two typical strains, representing the different lifestyles of this species, were evaluated for comparison: Symbioflor 1 and the pathogen V583. To the best of our knowledge, researchers have yet to investigate the influence of putative pathogenic or probiotic *E. faecalis* on human placental trophoblast cells by comparative transcriptomic analysis.

Our invasion assays demonstrated that both the pathogenic V583 strain and the probiotic Symbioflor 1 strain could adhere and internalize into human placental trophoblast cells. Furthermore, regardless of their diverse origins and lifestyles, these strains were found to show similar *in vitro* invasion ability. This observation was also consistent with our recent study of *E. faecalis* OG1RF in which 6.32 ± 0.10 Log_10_ CFU/mL of bacteria were seen to invade and 2.23 ± 0.29 Log_10_ CFU/mL were seen to internalize [8]. Similarly, Peng et al. found that the same three *E. faecalis* strains showed a similar adhesion rate but exhibited a different translocation rate in Ptk6 epithelial cell monolayers [15]. Bierne et al. reported that *E. faecalis* has the ability to internalize into intestinal LoVo cells [16]. These results demonstrated that different *E. faecalis* strains are able to pass through both intestinal and placental barriers, and their differences in invasion ability may be related to the cell model selected.

The influence of *E. faecalis* on human placental trophoblast cells was further investigated using DNA microarray analysis. We previously illustrated the biological effects and associated molecular mechanisms of *E. faecalis* OG1RF on placental function using a BeadChip microarray [8]. DNA microarrays have a distinct advantage over other techniques as they can provide both qualitative and quantitative data for a vast numbers of DEGs with high levels of sensitivity [17]. Li et al. used a DNA microarray to compare gene expression patterns between untreated and Aa-LPS-treated BeWo cells [18]. The changes in gene expression in human trophoblasts that pose a direct impact to placental and fetal health are now recognized as biomarkers [19]. According to the comparative results of DNA microarray analysis, the total number of DEGs appeared to be similar for both Symbioflor 1 and V583 treatment groups. Furthermore, the number of genes identified was slightly higher than that previously found in *E. faecalis* OGIRF [8]. However, almost half of the DEGs (both upregulated and downregulated) were unique to each group suggesting that different strains exhibit different effects on the placenta. According to our KEGG results, the V583 strain exhibited a greater number of signaling pathways than the Symbioflor 1 strain, thus indicating that pathogenic *E. faecalis* may in particular cause a stronger response in BeWo cells. Moreover, apoptosis, ErbB, B cell receptor, and leukocyte transendothelial migration signaling pathways were only activated in the V583 group. Similarly, pathogen-induced apoptosis in human placental trophoblasts can also lead to septicemia during pregnancy [18]. A previous study showed that the B cell receptor signaling pathway and leukocyte transendothelial migration were also found in the host cell infected by *Streptococcus pneumoniae* [20]. In particular, PPAR (peroxisome proliferator-activated receptor delta) is critically essential for placental development and function. This typical nuclear receptor has also been suggested to increase the placental fatty acid uptake [21]. Furthermore, studies have shown that PPAR-*δ*-deficiency mice offspring can lead to growth retardation and impairment of neural development [22]. Our present results showed that PPARD was upregulated in the Symbioflor 1 treatment group but downregulated in the V583 treatment group. These results suggest that the presence of pathogenic *E. faecalis* may stunt fetal and placental growth and development. This result is in high agreement with our previous studies showing that pregnant mice given oral doses of *E. faecalis* OG1RF exhibit changes in terms of their fetal and placental growth and development [7, 8]. Moreover, both ADAM10 and APAF1 were exclusively upregulated in the V583 treatment group but not in the Symbioflor 1 treatment group. Previous studies have demonstrated that ADAM10 can mediate E-cadherin shedding and regulate epithelial cell-cell adhesion, thus exerting a direct impact on early embryonic development *in vivo* [23]. APAF1 is considered as a key player in apoptosis during embryonic development [24]. We also found that CAPN2, POFUT1, and GAS1 were all downregulated in the V583 treatment; these proteins have been shown to play an important role in embryonic development [25–27]. In particular, mouse embryos lacking Pofut1 have been shown to exhibit defects in their cardiovascular system [26]. Martinelli et al. found that Gas1 mutant pups were only 3/4 the size of their control littermates [27] and showed similar abnormalities as fetal mice infected with *E. faecalis* OG1RF [7].

## 5. Conclusions

Our present analysis identified that a large proportion of transcriptional responses in BeWo cells differed when compared between infection caused by pathogenic and probiotic *E. faecalis*. Several unique DEGs and signal pathways were identified in the two strains. These data constitute a strong basis for understanding the mechanisms underlying the differential effects caused by pathogenic and probiotic strains of *E. faecalis*.

## Figures and Tables

**Table 1:** *Enterococcus faecalis* strains used in this study.

Strain	Number	Country	Source	Isolation	Characteristics	References
Symbioflor 1	DSM 16431	Germany	Nonhospitalized person	Feces	Probiotic	[11]
V583	ATCC 700802	USA	Hospitalized patient	Blood	Pathogen, Ery^R^, Gen^R^, and Van^R^	[12]

^*∗*^Ery, erythromycin; Gen, gentamicin; Van, vancomycin; R_,_ resistance.

**Table 2:** DEGs containing relevant terms of the signal pathway generated by KEGG pathway analysis.

Group	Term	Genes
Symbioflor 1 group	hsa04010: MAPK signaling pathway	FGFR4, PDGFB, GNA12, PPM1A, MKNK2, PPM1B, MAP3K7, MAX, FOS, MAP3K5, MAP3K3, HSPA7, MAP2K7, MYC, HSPA8, RASA2, EGFR, TAOK1, TGFBR2, DUSP5, ATF4, RPS6KA4, MAPK13, MAPK14, JUN, RAP1A, PLA2G6, MAPK7, GADD45B, GADD45A, PLA2G2D (31)
	hsa04630: Jak-STAT signaling pathway	PTPN6, CRLF2, LEPR, CBL, SOCS4, BCL2L1, SOCS5, IL7R, IL10, STAT3, LEP, GH2, ZFP91, STAT4, SPRY1, EP300, IL10RB, IL5RA, SPRED1, MPL, MYC, PIK3R1 (22)
	hsa04520: adherens junction	EGFR, LOC646821, PTPN6, BAIAP2, TGFBR2, CTNND1, ACP1, WAS, CTNNB1, VCL, MAP3K7, PVRL4, EP300, PVRL1, SORBS1, PVRL2 (16)
	hsa04660: T cell receptor signaling pathway	PTPN6, NFKBIB, CBL, MALT1, IL10, MAP3K7, FOS, NCK2, MAPK13, MAPK14, PAK4, JUN, ZAP70, MAP2K7, PIK3R1 (15)
	hsa04115: p53 signaling pathway	ZMAT3, RPRM, SESN2, CCNG2, CDK2, CCNB1, PPM1D, CDKN1A, CDKN2A, BAX, RRM2, GADD45B, GADD45A (13)
	hsa05130: pathogenic *Escherichia coli* infection	LOC646821, NCK2, YWHAZ, KRT18, LOC399942, TUBA3E, WAS, ITGB1, CTNNB1, TTLL3 (10)

V583 group	hsa04010: MAPK signaling pathway	FGFR1, FGF18, PDGFB, MRAS, PPP3R1, PPM1A, MKNK2, CACNB3, GNG12, PPM1B, NFKB2, FOS, MAP3K5, CASP3, NFATC4, MYC, HSPA8, RASA2, EGFR, NTF4, TAOK1, TGFBR2, DUSP5, ATF4, DUSP2, JUN, GADD45G, RAP1A, MAPK9, GADD45B, PLA2G3, PLA2G2D, GADD45A (33)
	hsa04670: leukocyte transendothelial migration	ACTB, F11R, LOC646821, NCF4, SIPA1, CTNND1, ITGB1, ITGAM, CTNNB1, VCL, PTK2, CYBB, PTK2B, RAP1A, PIK3R3, PIK3R1, LOC284620 (17)
	hsa04115: p53 signaling pathway	ZMAT3, SESN2, CCNG2, CDK2, CCNE2, PPM1D, CDKN1A, CASP3, CDKN2A, RRM2, GADD45G, BAI1, APAF1, MDM4, GADD45B, GADD45A (16)
	hsa04660: T cell receptor signaling pathway	BCL10, PTPN6, NFKBIE, NFKBIB, CBL, PPP3R1, IL10, FOS, NCK2, JUN, PAK4, MAPK9, NFATC4, PIK3R3, PIK3R1, NFATC1 (16)
	hsa04210: apoptosis	IAP, AIFM1, PPP3R1, BAD, BCL2L1, CAPN2, CASP6, TNFSF10, CASP3, RIPK1, PRKAR1B, IL1RAP, APAF1, PIK3R3, PIK3R1 (15)
	hsa04012: ErbB signaling pathway	EGFR, CBL, BAD, NCK2, PTK2, CDKN1A, CDKN1B, PAK4, JUN, GAB1, MAPK9, PIK3R3, MYC, PIK3R1 (14)
	hsa04520: adherens junction	ACTB, EGFR, LOC646821, FGFR1, PTPN6, TGFBR2, CTNND1, ACP1, VCL, CTNNB1, PVRL4, EP300, PVRL1, PVRL2 (14)
	hsa04662: B cell receptor signaling pathway	BCL10, PTPN6, IFITM1, NFKBIE, NFKBIB, PPP3R1, FOS, JUN, NFATC4, PIK3R3, PIK3R1, BLNK, NFATC1 (13)
	hsa05130: pathogenic *Escherichia coli* infection	ACTB, LOC646821, NCK2, KRT18, ARPC3, LOC399942, TUBA3E, ITGB1, CTNNB1, TTLL3 (10)

**Table 3:** DEGs classified into placenta and embryonic development of the GO biological process category.

Group	Term	Genes
Symbioflor 1 group	GO: 0001701∼in utero embryonic development	MAFG, MAFF, XRCC2, GABPA, GNA12, SPINT1, EGLN1, BCL2L1, MBNL1, ITGB1, TPM1, CITED2, HES1, NDEL1, MSX1, PSMC4, HAND1, HSF1, TEAD4, PKD1, MKL2, LOC652826 (22)
	GO: 0001890∼placenta development	VWF, PPARD, HSF1, HAND1, CCNF, PLCD3, SPINT1, HS6ST1, EGLN1, PRDX3, CITED2 (11)
	GO: 0001892∼embryonic placenta development	HSF1, HAND1, SPINT1, EGLN1, CITED2 (5)
	GO: 0060136∼embryonic process involved in female pregnancy	HSF1, CITED2 (2)

V583 group	GO: 0043009∼chordate embryonic development	GNA13, SYVN1, XRCC2, NDST1, EGLN1, BCL2L1, ITGB1, TPM1, CITED2, HSF1, PKD1, MKL2, FLVCR1, BCL10, MAFF, ADAM10, ZNF830, TGFBR2, SPINT1, MBNL1, LIG4, GAS1, CAPN2, BRCA1, HES1, DLX2, TULP3, EP300, TSC1, PSMC4, SP3, HOXB6, MNX1, TGIF1, RIPPLY1, FOXC1, APAF1, LOC652826, POFUT1 (39)
	GO: 0009792∼embryonic development ending in birth or egg hatching	Same as GO: 0043009∼chordate embryonic development (39)
	GO: 0001701∼in utero embryonic development	GNA13, XRCC2, SYVN1, EGLN1, BCL2L1, TPM1, ITGB1, CITED2, HSF1, PKD1, MKL2, FLVCR1, MAFF, ADAM10, ZNF830, SPINT1, LIG4, MBNL1, CAPN2, HES1, TULP3, PSMC4, SP3, FOXC1, LOC652826 (25)
	GO: 0001890∼placenta development	VWF, PPARD, CYP27B1, HSF1, SP3, CCNF, PLCD3, SPINT1, EGLN1, CITED2 (10)
	GO: 0048701∼embryonic cranial skeleton morphogenesis	DLX2, TULP3, NDST1, TGFBR2, GAS1 (5)
	GO: 0060136∼embryonic process involved in female pregnancy	HSF1, SP3, CITED2 (3)

## Data Availability

The datasets used and/or analyzed during the current study are available from the corresponding author upon request.

## Supplementary Materials

Supplementary Materials Table S1. Differential gene expression profile of BeWo cell in response to *Enterococcus faecalis* Symbioflor 1 invasion. Table S2. Differential gene expression profile of BeWo cell in response to *Enterococcus faecalis* V583 invasion. Table S3. Differentially expressed genes affected by *Enterococcus faecalis* Symbioflor 1 classified with GO database. DEGs were characterized functionally by comparison against GO database and classified into three different categories, namely, the biological process, cellular component, and molecular function. Table S4. Differentially expressed genes affected by *Enterococcus faecalis* V583 classified with GO database. DEGs were characterized functionally by comparison against GO database and classified into three different categories, namely, the biological process, cellular component, and molecular function.
